# Saint Bartholomew's Hospital Reports

**Published:** 1892-12

**Authors:** 


*Saint Bartholomew's Hospital Reports*. Edited by W. S. Church, M.D., and W. J. Walsham, F.R.C.S. Vol. XXVII. Smith, Elder & Co. 1891.

This volume is not inferior in interest to its predecessors. Of the numerous papers contained therein, that by Dr. Vincent Harris "On the Treatment of Hæmoptysis" is worthy of especial mention, and an interesting essay "On the Prognosis of Simple Gastric Ulcer," by Dr. S. H. Habershon, presents the most recent views on a question in which reticence and wariness are the only safe course. The obituary notice of Dr. Robert Martin ranks him among the great men of his generation, and gives us a very interesting account of one who, when tried by sickness and disappointment set an example which others might endeavour humbly to follow.

